# *In vivo* Acquisition of Carbapenemase Gene *bla*_KPC-2_ in Multiple Species of *Enterobacteriaceae* through Horizontal Transfer of Insertion Sequence or Plasmid

**DOI:** 10.3389/fmicb.2016.01651

**Published:** 2016-10-21

**Authors:** Baixing Ding, Zhen Shen, Fupin Hu, Meiping Ye, Xiaogang Xu, Qinglan Guo, Minggui Wang

**Affiliations:** ^1^Institute of Antibiotics, Huashan Hospital, Fudan UniversityShanghai, China; ^2^Key Laboratory of Clinical Pharmacology of Antibiotics, Ministry of HealthShanghai, China

**Keywords:** Enterobacteriaceae, carbapenem resistance, insertion sequence, plasmid, KPC-2

## Abstract

**Objectives:** Current worldwide spread of carbapenem resistance in Enterobacteriaceae constitutes a critical public health threat. This study aims to investigate how carbapenem resistance is acquired in Enterobacteriaceae in patients during antimicrobial therapy.

**Methods:** Clinical strains from the same anatomical site of the same patients that converted from carbapenem-susceptible to resistant during antimicrobial therapy and showed identical or similar PFGE patterns were identified. The similarly sized plasmids carried by the susceptible and resistant strains, the latter containing the carbapenemase genes, were sequenced and analyzed.

**Results:** Paired strains were identified from four patients: three had neurosurgical conditions while the other had acute exacerbation of COPD. Two pairs of *Klebsiella pneumoniae* (KP1-S/R and KP2-S/R, S and R indicating susceptible and resistant strains, respectively), one pair of *Morganella morganii* (MM-S/R) and one pair of *Enterobacter aerogenes* (EA-S/R) were collected. All four carbapenem-resistant strains carried plasmids harboring *bla*_KPC−2_. Compared with the similarly sized plasmids in KP1-S and KP2-S, an insertion sequence that includes IS*Kpn6*-like, *bla*_KPC−2_ and IS*Kpn8* was noted in pKP1-R and pKP2-R. Strains MM-R and EA-R had *bla*_KPC−2_-carrying plasmids not resembling plasmids in strains MM-S and EA-S suggesting their new acquisition while on therapy.

**Conclusions:** Enterobacteriaceae can acquire carbapenem resistance during antimicrobial therapy through horizontal transfer of an insertion sequence or plasmid.

## Introduction

Carbapenems are the most effective antibiotics for the treatment of refractory Gram-negative bacterial infections. And yet, carbapenem-resistant Enterobacteriacea*e* (CRE) are increasingly isolated from clinical samples globally (Nordmann et al., 2012). According to the CHINET (a national antimicrobial resistance surveillance project in China), the rates of carbapenem-resistant *K. pneumoniae* was 2.4% in 2005 and increased to approximately 13.4% in 2014 (Hu et al., 2016). The treatment options for CRE are limited and these infections are associated with poor clinical outcome (Schwaber et al., 2008). Understanding how CRE spreads is crucial for timely implementation of infection control measures.

The most common mechanism of carbapenem resistance in Enterobacteriaceae is the production of plasmid-mediated carbapenemases, and KPCs (*Klebsiella pneumoniae* carbapenemases) is currently the most prevalent in many countries including China (Tzouvelekis et al., 2012). Horizontal transfer through transposons and integrons may account for the dissemination of carbapenemase genes carried by CRE strains (Munoz-Price and Quinn, 2009). Interspecies transfer of the gene encoding carbapenemase OXA-48 has been demonstrated in the gut using animal models, suggesting that horizontal transfer of carbapenemase genes may be an important mode of spread of CRE (Göttig et al., 2015).

Previously, we have reported the transfer of NDM-1 gene in *E. coli* in a patient: a clonally related carbapenem-resistant *E. coli* strain was isolated 1 month later after the isolation of a carbapenem-susceptible *E. coli* (Ding et al., 2015). A similar observation has also been made with *K. pneumoniae* in a patient with pneumonia (Elliott et al., 2006). It is also reported that, in some patients with Gram-negative bacterial infections, bacterial strains belonging to same species from the same anatomical site might lose susceptibility to the agents that are being used for treatment (Lee et al., 2011; Solé et al., 2015).

Numerous studies have addressed epidemiology, risk factors and treatment outcome of CRE infections (Tzouvelekis et al., 2012), but relatively little attention has been paid to the genetic process of conversion from carbapenem-susceptible to resistant of a bacterial strain. In this study, we retrospectively surveyed the clinical microbiology data of Enterobacteriaceae isolates and the clinical data of patients in a tertiary hospital in Shanghai, China from January 2011 to December 2012. As a result, four patients who had Enterobacteriaceae strains with identical or similar PFGE patterns and conversion of carbapenem susceptibility from susceptible to resistant during antimicrobial therapy were identified. We revealed that horizontal transfer of an insertion sequence or plasmid carrying *bla*_KPC−2_ was responsible for the *in vivo* development of carbapenem resistance.

## Methods

### Patients

The study was conducted at Huashan Hospital, Fudan University, a 1300-bed tertiary care hospital in Shanghai, China. The clinical microbiology data of Enterobacteriaceae strains from inpatients between January 2011 and December 2012 were retrospectively reviewed. Two or more strains of identical or similar species isolated from the same anatomical site of a patient were collected if their carbapenem susceptibility converted from susceptible to resistant during antimicrobial therapy. Isolates from the same patients with identical or similar PFGE patterns were then further studied. The clinical data were collected, including general demographics, such as age and sex; the ward to which the patient was assigned after admission; previous use of antimicrobial agents, particularly carbapenems, extended spectrum cephalosporins and fluoroquinolones. This study was approved by the Institutional Review Board of Huashan Hospital and was exempted for obtaining written informed consents from patients (number: KY2014-233). Patient records were anonymized and de-identified prior to analysis.

### Clinical strains and susceptibility testing

All the strains were identified by the Vitek 2 automated system (BioMerieux, France). Antimicrobial susceptibility testing was performed by the agar dilution method as recommended by the Clinical and Laboratory Standards Institute (CLSI, 2014). Isolates with nonsusceptibility to either of the two carbapenems (imipenem and meropenem) tested were classified as carbapenem-resistant.

### Carbapenemase phenotypic detection

Modified Hodge Test (MHT) was used for carbapenemase detection, and the positive result has a clover leaf-like indentation of the *E. coli 25922* growing along the test organism growth streak within the disk diffusion zone. The phenotypic detection of carbapenemase was also evaluated with boronic acid disk tests, the test was considered positive for class A carbapenemase production when the diameter of the growth-inhibitory zone around imipenem disk with boronic acid was ≥5 mm larger than that around a disk containing imipenem alone.

### PFGE analysis

PFGE was employed to determine the genetic relationship among the clinical isolates of the same species from the same patients. Genomic DNA was digested with *Xba*I for 4 h at 37°C. The digested DNAs were then separated in 1% agarose gel using a CHEF-DRIII system (Bio-Rad Laboratories), with a linear pulse time ramped from 2 to 70 s for 24 h at 14°C and constant voltage of 6 V/cm. Similarity between PFGE patterns was determined according to the criteria proposed by Tenover et al. and with Bionumerics software using the Dice coefficient. Strains sharing the restriction patterns of less than or equal to three-band differences (Tenover et al., 1995) and similarity coefficients >80% were considered to be closely related strain type (Harris et al., 2007; Wu et al., 2014).

### PCR detection of β-lactamase genes

PCR detection of β-lactamase genes encoding carbapenemases (*bla*_AIM_, *bla*_BIC_, *bla*_GIM_, *bla*_IMP_, *bla*_KPC_, *bla*_NDM_, *bla*_OXA−48_, *bla*_SIM_,*bla*_SPM_, and *bla*_VIM_) (Poirel et al., 2011), extended-spectrum β-lactamases (*bla*_CTX−M_) (Woodford et al., 2006), plasmid-mediated AmpC β-lactamase (*bla*_ACC_, *bla*_CIT_, *bla*_DHA_, *bla*_EBC_, *bla*_FOX_, and *bla*_MOX_) (Pérez-Pérez and Hanson, 2002) was performed as previously described. PCR amplicons were sequenced and compared with sequences available in the GenBank database using BLAST searches.

### Plasmid analysis

Plasmid DNA was extracted using a Plasmid Midi Kit (QIAGEN, Germany) according to the manufacturer's instructions. The DNA was electrophoresed in 0.8% agarose for 1.5 h. Three reference strains carrying different sizes of plasmids, with several plasmids in *E. coli* V517 (size, 54, 5.6, 5.1, 3.9, 3.0, 2.7, and 2.1 kb), R1 (92 kb) in *E. coli* J53 and R27 (182 kb) in *E. coli* J53. The plasmids of clinical strains were introduced by electroporation into *E. coli* DH10B using a Gene Pulser II system (Bio-Rad Laboratories). Putative transformants were selected through PCR screening for *bla*_KPC_ from the colonies growing on Mueller-Hinton agar plates containing ampicillin (50 μg/mL). Transformants harboring a single plasmid with *bla*_KPC_ were selected. For carbapenem susceptible strains, transformants with plasmids of similar sizes with the corresponding *bla*_KPC_-positive plasmids were selected for further investigation. PCR-based replicon typing (Carattoli et al., 2005) was performed on the plasmid of these transformants. Each plasmid was then subjected to EcoRI digestion for 2 h at 37°C and electrophoresed on a 0.8% agarose gel for 1.5 h.

Southern blot analysis was performed with DIG High Prime DNA Labeling and Detection Starter Kit II (F. Hoffmann-La Roche Ltd., Basel, Swiss). Plasmids were separated on a 0.8% agarose gel and transferred to a nylon membrane as described previously (Southern, 1975). The probe for *bla*_KPC−2_ was amplified using KPC-Fm/KPC-Rm primers (Poirel et al., 2011) using KP1-R as a template. Following gel-purification, the 800-bp product was randomly primed with DIG-11-dUTP using the DIG-High Prime DNA Labeling and Detection Starter Kit II.

### High-throughput sequencing

Plasmid DNA concentration was assessed using Qubit 2.0 Fluorometer (Life Technologies, Carlsbad, CA, USA). Ten nanograms of DNA was employed to prepare barcoded libraries with the Ion Plus Fragment Library kit (Life Technologies). Template preparation and enrichment was performed with the Ion OneTouch^TM^ 2 System (Life Technologies). Finally, sequencing was carried out using Ion 318TM chips on the Ion PGM System (PGM^TM^, Life Technologies) and with the Ion PGM^TM^ Sequencing 400 kit. Sequence gaps were filled by PCR and sequenced using primer walking. Lasergene sequence analysis software system was used to align the sequence.

### Nucleotide sequence accession numbers

The annotated sequences of pKP1-S, pKP1-R, pKP2-S, and pKP2-R have been submitted to GenBank nucleotide sequence database under accession numbers KT922275, KT922274, KT922273, and KT922272, respectively.

## Results

### Clinical cases

From January 2011 to December 2012, four pairs of Enterobacteriaceae from four patients with identical species, identical or similar PFGE patterns and discordant carbapenem susceptibility were identified. Of these four patients, two had a pair of *K. pneumoniae*, one a pair of *M. morganii* and the other a pair of *E. aerogen*es (Table 1).

**Table 1:** **Clinical and microbiological characteristics of four pairs of isolates with conversion of carbapenem susceptibility**.

**Age (year)/sex**	**Patient 1**		**Patient 2**		**Patient 3**		**Patient 4**	
	**61/M**		**63/M**		**96/M**		**52/M**	
Strain	*K. pneumoniae* 1-S	*K. pneumoniae* 1-R	*K. pneumoniae* 2-S	*K. pneumoniae* 2-R	*M. morganii*-S	*M. morganii*-R	*E. aerogenes*-S	*E. aerogenes*-R
Time from hospitalization to bacterial isolation (d)	6	29	224	231	36	129	5	17
Antibiotics use before bacterial isolation^a^	CXM	CXM; CED	CAZ; AK; SCF	CAZ; LEV	/	MEM	/	TGC; SCF
Source	Urine	Urine	Sputum	Sputum	Sputum	Sputum	Sputum	Sputum
ST^b^	11	11	11	11	NA	NA	NA	NA
*Minimum inhibitory concentrations* (μg/ml)								
Imipenem	0.25	8	0.125	32	1	2	0.5	16
Meropenem	0.5	16	0.25	32	≤ 0.06	0.25	≤ 0.06	16
Piperacillin/tazobactam	>128	>128	>128	>128	0.25	16	8	128
Ceftazidime	16	32	128	128	8	16	8	64
Amikacin	>128	>128	>128	>128	1	1	>128	>128
Levofloxacin	128	64	128	128	4	4	2	4
Polymyxin B	1	1	1	1	1	1	1	1
Tigecycline	1	1	2	2	2	2	2	2

a CXM, cefuroxime; CED, cefradine; CAZ, ceftazidime; AK, amikacin; SCF, cefoperazone-sulbactam; MEM, meropenem; TGC, tigecycline; LEV, levofloxacin.

b NA, not applicable.

Patient 1. A 61-year-old male with cerebral hemorrhage presented to the emergency department in March 2011. After hematoma cleaning, he developed catheter-associated urinary tract infection. The patient was empirically treated with intravenous cefuroxime for 2 weeks with resolution of fever, then switched to oral cefradine. Urine culture on hospital day 6 grew carbapenem-susceptible *K. pneumoniae* (KP1-S). Carbapenem-resistant *K. pneumoniae* (KP1-R) was isolated from the urine on day 30. No other multidrug-resistant isolates were isolated after this.

Patient 2. A 63-year-old male was hospitalized with meningioma in October 2010. After resection of meningioma and placement of tracheostomy, he was diagnosed with esophageal injury and possible tracheoesophageal fistula. He had pneumonia 1 month later, and carbapenem-susceptible *Pseudomonas aeruginosa* and MDR *Acinetobacter baumannii* were isolated from sputum. The patient was treated with ceftazidime, amikacin and cefoperazone-sulbactam. Seven and half months later, carbapenem-susceptible *K. pneumoniae* (KP2-S) was isolated from the sputum, then he received ceftazidime and levofloxacin. One week after the therapy, carbapenem-resistant *K. pneumoniae* (KP2-R) grew from the sputum.

Patient 3. A 96-year-old male was admitted to the ICU in March 2011 because of acute exacerbation of chronic obstructive pulmonary disease (COPD). He had been colonized with multiple MDR organisms in the respiratory tract, including carbapenem-resistant *P. aeruginosa* and carbapenem-resistant *K. pneumoniae*. A carbapenem-susceptible *M. morganii* (MM-S) was isolated from the sputum a month later. Hospital-acquired pneumonia was presumed and treated with meropenem for 2 months. Four months after admission, a carbapenem-resistant *M. morganii* (MM-R) was detected in the sputum.

Patient 4. A 52-year-old male was admitted after sustaining craniocerebral injuries from a fall in November 2012. He required evacuation of intracranial hematoma and decompressive craniotomy. Five days after admission, a carbapenem-susceptible *E. aerogenes* (EA-S) was isolated from the sputum. During the first 2 weeks of hospitalization, a variety of MDR organisms were isolated, including MDR *A. baumannii* (respiratory tract), carbapenem-resistant *P. aeruginosa* (respiratory tract) and carbapenem-resistant *K. pneumoniae* (blood). He had intermittent fever during hospitalization. The patient was treated with tigecycline and cefoperazone-sulbactam. Seventeen days after admission, a carbapenem-resistant *E. aerogenes* (EA-R) strain was isolated from the sputum.

### Characterization of clinical strains

Antimicrobial susceptibility testing revealed that strains KP1-R, KP2-R, MM-R, EA-R were carbapenem-non-susceptible with imipenem MICs of 8, 32, 2, and 16 mg/L, respectively (Table 1). The modified Hodge test was positive for carbapenemase production and synergy between boronic acid and imipenem was observed, suggesting the presence of KPC carbapenemase. The *bla*_KPC−2_ gene was detected by PCR in all the four carbapenem non-susceptible strains. No other carbapenemase genes were detected (Table 2).

**Table 2:** **Comparison of KPC-2 plasmid and plasmid from corresponding carbapenem-susceptible strain and of minimal inhibitory concentration of transformants**.

**Transformants^a^**	**KP1-S**	**KP1-R**	**KP2-S**	**KP2-R**	**MM-S**	**MM-R**	**EA-R**
Plasmid	pKP1-S	pKP1-R	pKP2-S	pKP2-R	pMM-S	pMM-R	pEA-R
Plasmid size (approximately kb)	≈140	≈140	≈170	≈170	≈170	≈150	≈40
Inc group	IncFIIk	IncFIIk	IncFII; IncR	IncFII; IncR	IncA/C2	IncFII; IncN	Non-typable
β-lactamase genes in plasmid	TEM-1;	KPC-2 TEM-1;	TEM-1;	KPC-2 TEM-1;	TEM-1;	KPC-2	KPC-2
	CTX-M-9	CTX-M-9;	SHV-11;	SHV-11;	CTX-M-9		
*Minimum inhibitory concentrations* (μg/ml)							
Imipenem	≤0.06	0.125	≤0.06	0.25	≤0.06	1	0.5
Ertapenem	≤0.06	0.25	≤0.06	0.25	≤0.06	2	0.5
Piperacillin/tazobactam	4	64	2	64	4	>128	>128
Ceftazidime	2	4	32	32	1	32	>128
Amikacin	1	1	>128	>128	4	2	1
Levofloxacin	≤0.06	≤0.06	≤0.06	≤0.06	≤0.06	≤0.06	≤0.06

a KP, Klebsiella pneumoniae; MM, Morganella morganii; EA, Enterobacter aerogenes.

**Figure 1:**
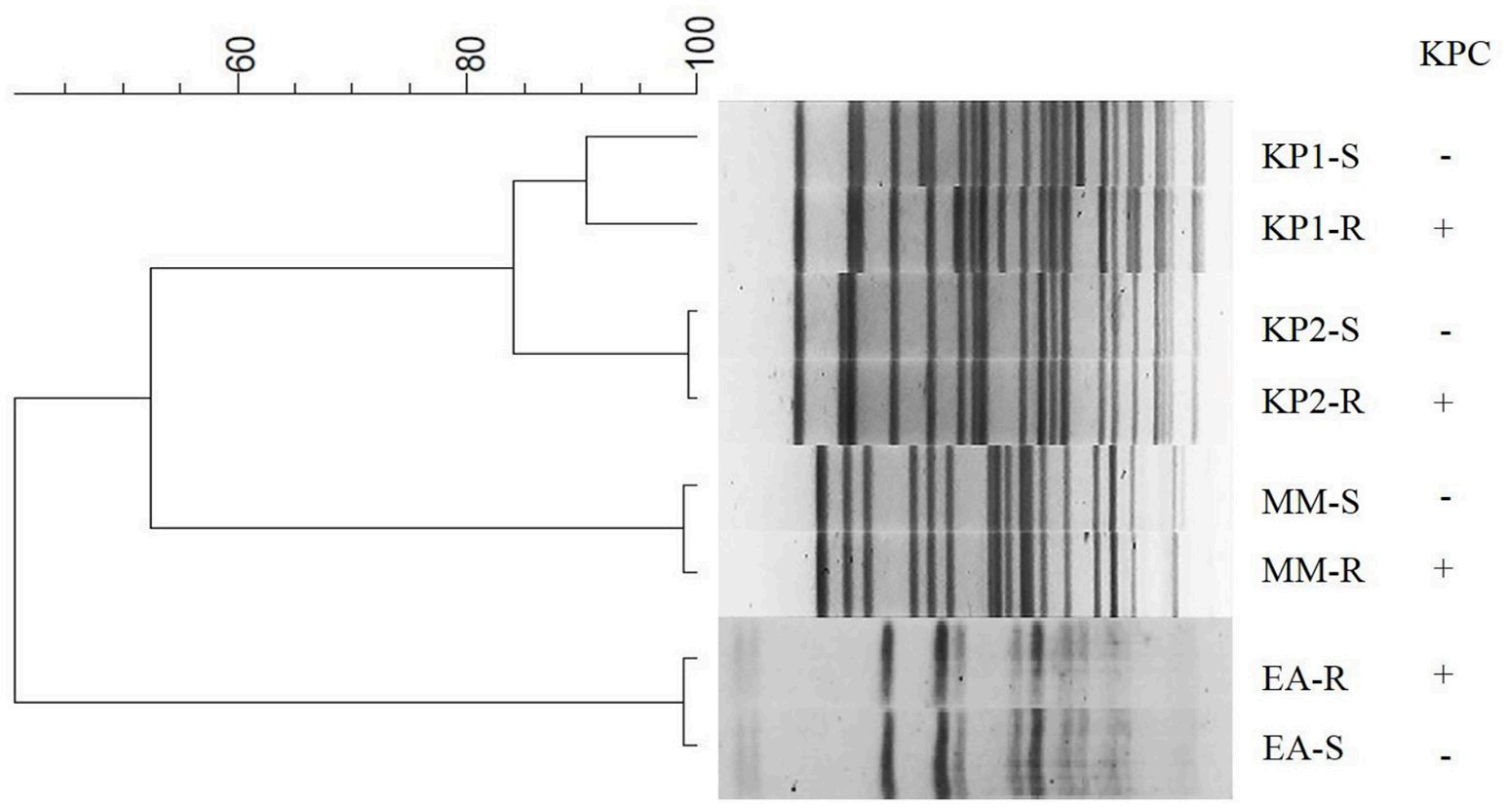
**Dendrogram of XbaI-digested genomic DNA of clinical isolates**. The dendrogram was constructed using BioNumerics software version 6.6 (Applied Maths, St-Martens-Latem, Belgium). KP, *Klebsiella pneumoniae*; MM, *Morganella morganii*; EA, *Enterobacter aerogenes*.

Three pairs of Enterobacteriaceae (KP2-S and KP2-R, MM-S and MM-R, EA-S, and EA-R) showed identical PFGE patterns, suggesting that the two strains from each patient belonged to the same clone. The pair of *K. pneumoniae* 1 (KP1-S and KP1-R) strains had >90% similarity of PFGE profiles, indicating they were highly related (Figure 1). Plasmid DNA analysis revealed that similar plasmid profiles were found for the pairs of *K. pneumoniae* isolates (KP1-S and KP1-R, KP2-S, and KP2-R). The plasmid profile was identical between *E. aerogenes* strains EA-S and EA-R except that EA-R had one extra plasmid. Similarly sized plasmids also existed in *M. morganii* strains MM-S and MM-R (Supplementary Figure 1). Southern blot revealed that *bla*_KPC−2_ was located on plasmids in all four carbapenem-non-susceptible strains (KP1-R, KP2-R, MM-R, and EA-R) (Supplementary Figure 1).

### Comparison of plasmids between carbapenem-resistant and -susceptible strains

In order to compare the plasmids harboring *bla*_KPC−2_ in carbapenem-resistant isolates, they were isolated through electroporation experiment and designated pKP1-S, pKP1-R, pKP2-S, pKP2-R, pMM-S, pMM-R, and pEA-R.

The screening of β-lactamase genes revealed that the pair of plasmids pKP1-S and pKP1-R, shared the same resistance genes *bla*_TEM−1_ and *bla*_CTX−M−9_, except that *bla*_KPC−2_ was detected in pKP1-R. pKP2-S harbored *bla*_TEM−1_ and *bla*_SHV−11_, while *bla*_KPC−2_, *bla*_TEM−1_, and *bla*_SHV−11_ were carried by pKP2-R. pMM-S harbored *bla*_TEM−1_ and *bla*_CTX−M−9_, while only *bla*_KPC−2_ was detected by pMM-R. *bla*_KPC−2_ was detected in pEA-R. (Table 2).

Plasmids from two pairs of transformants (pKP1-S and pKP1-R; pKP2-S and pKP2-R) yielded similar EcoRI digestion patterns. EcoRI digestion patterns of pMM-S and pMM-R were inconsistent (Supplementary Figure 1, Table 2).

### Sequence analysis

Three pairs of plasmids (pKP1-S, pKP1-R, pKP2-S, pKP2-R, pMM-S, and pMM-R) were sequenced to reveal the critical processes involved in the acquisition of carbapenemases. The preliminary replicon types of plasmids were determined with the replicon-specific primers, but pMM-R and pEA-R could not be typed. Thus, these six plasmids were further typed with sequence alignment based on the plasmid sequencing data.

**Figure 2:**
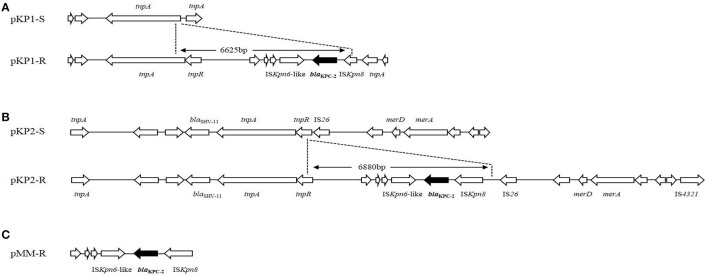
**Plasmid structures surrounding *bla*_KPC−2_ gene in *K. pneumoniae and M. morganii***. **(A)** Comparison between plasmids pKP1-S and pKP1-R. **(B)** Comparison between plasmids pKP2-S and pKP2-R. **(C)** *bla*_KPC−2_ and its surrounding genetic elements of plasmid pMM-R. The differences between the plasmids are shown, and the genes are depicted as arrows according to the direction of transcription. The dots indicated the inserted DNA sequences in pKP1-R and pKP2-R. The *bla*_KPC−2_ genes are shown in black.

IncFIIk replicon was detected in plasmid pKP1-S and pKP1-R. The skeleton of plasmid pKP1-R was similar with that of pKP1-S. A 2.6 kb scaffold in pKP1-S was not present in pKP1-R, while two scaffolds about 2.3 and 2.4 kb in pKP1-R were not found in pKP1-S. Plasmid pKP1-R was very similar to two *bla*_KPC−2_-carrying plasmids, pKP048 (GenBank accession no. FJ628167.2) and pKPC-LK30 (GenBank accession no. KC405622.1). Analysis on an 11,954-bp scaffold carrying *bla*_KPC−2_ gene in pKP1-R (it is about 6 kb larger than the corresponding scaffold in pKP1-S, GenBank accession no. KT922274), we found that a 6625-bp DNA fragment inserted at the upstream end of *tnpA* had taken place on pKP1-S (GenBank accession no. KT922275). The *bla*_KPC−2_ gene was located on an insertion sequence which included an IS*Kpn6*-like element followed by *bla*_KPC−2_ and interrupted IS*Kpn8* (Figure 2).

Both IncFII and IncR replicons were present in pKP2-S and pKP2-R. The scaffold of plasmid pKP2-R was also similar with that of pKP2-S. Two fragments about 4.2 and 1.3 kb in pKP2-S were not present in pKP2-R. Over 80% sequence of plasmid pKP2-R is identical to pKPC-LK30 (GenBank accession no. KC405622.1). For pKP2-R, a nucleotide sequence of 25,428 bp surrounding *bla*_KPC−2_ gene was obtained (GenBank accession no. KT922272). Compared with the scaffold obtained from pKP2-S (GenBank accession no. KT922273), a DNA fragment insertion of 6880 bp was found. Like pKP1-R, the insertion sequence including the IS*Kpn6*-like element, *bla*_KPC−2_ and IS*Kpn8* was inserted at the upstream end of *tnpR* and downstream of IS26 in pKP2-S (Figure 2). A 5819-bp common region in the inserted DNA sequences from pKP1-R and pKP2-R is identical to Tn*1721* that includes IS*Kpn6*-like, *bla*_KPC−2_ and interrupted IS*Kpn8.* The *bla*_KPC−2_ was also carried by the same insertion sequence including IS*Kpn6*-like, *bla*_KPC−2_ and IS*Kpn8* in plasmid pMM-R (Figure 2).

IncA/C2 replicon was detected in plasmid pMM-S. A 120-kb region in pMM-S was similar to pVC1447 (GenBank accession no: KJ817377), a plasmid originally isolated from *Vibrio cholerae*. Both IncFII and IncN replicons were present in pMM-R. Plasmid pMM-R was 150 kb in length and had a 45-kb region identical to pKP1034 isolated from *K. pneumoniae* sequence type 11, which co-harbored *bla*_KPC−2_, *fosA3, rmtB* and *bla*_CTX−M−65_ (GenBank accession no: KP893385). No similar sequence was found between the scaffold of pMM-R and pMM-S.

## Discussion

In this study, we identified four instances where patients were initially colonized or infected with a *bla*_KPC_-negative strain, which later evolved to acquire *bla*_KPC_. These patients shared in common critical illness and recent receipt of β-lactam agents. However, only one patient received carbapenem therapy before the carbapenem-resistant clinical strains were isolated. As previously reported, underlying medical conditions and antimicrobial exposure (fluoroquinolones and antipseudomonal penicillins in particular) are important risk factors for CRE acquisition (Falagas et al., 2007; Schwaber et al., 2008). This study also underscores that β-lactam agents other than carbapenems may also exert selective pressure for acquisition of *bla*_KPC_ thus evolution to CRE.

In all four patients studied here, carbapenem resistance was associated with *in vivo* acquisition of an insertion sequence that carries *bla*_KPC−2_ or a plasmid harboring *bla*_*KPC*−2_ in Enterobacteriaceae. The most prevalent carbapenemase is KPC in China (Hu et al., 2012). The two pairs of *K. pneumoniae* belonged to ST11 and the four plasmids pKP1/2-R/S from these isolates were all classified as IncF family. ST11 was previously reported as the dominant clone in the region (Qi et al., 2011), and IncF family is the most predominant *bla*_KPC−2_-carrying plasmid, which is particularly well-suited for *K. pneumoniae* and for sustaining the presence of *bla*_KPC−2_ through multiple independent insertion and transposition events (Mathers et al., 2015).

Plasmids analysis provides insights into the mechanism of acquisition of genetic determinants carrying carbapenemase genes. Sequencing data revealed that pKP1-R and pKP2-R acquired mobile genetic elements containing *bla*_KPC−2_ gene. The two insertion sequences were parts of Tn*1721* as previously described (Shen et al., 2009). We also found that *tnpA* was located upstream of *bla*_KPC−2_ in pKP1-R and pKP2-R, suggesting the transposition might involve mobilization of the insertion sequence. Interestingly, the insertion sequences, together with the flanking regions of *bla*_KPC−2_ in plasmids pKP1-R and pKP2-R, are identical with other *bla*_KPC−2_ harboring plasmids pKP048 (Shen et al., 2009) and pKPC-LK30 (Chen et al., 2014), respectively. This finding suggests that insertion of the *bla*_KPC−2_ region into IncF family plasmids is likely to be site-specific. The differences of scaffolds between pKP1-R and pKP1-S, pKP2-R and pKP2-S indicate that DNA fragments exchange may take place. Further studies are still needed to elucidate the mechanisms of the DNA fragment mobilization. To the best of our knowledge, this is the first report of insertion of *bla*_KPC−2_-carrying genetic element to plasmids in clinical isolates of *K. pneumoniae in vivo.*

The sizes of plasmids pMM-S and pMM-R were similar but they had different restriction patterns (data not shown). pMM-S was belonged to plasmid type of IncA/C2, while pMM-R was IncFII and IncN. Plasmid sequencing data showed that no similar sequence was observed between the scaffold of pMM-S and pMM-R. A possible explanation is that the carbapenem-susceptible *M. morganii* acquired a phylogenetically distinct IncFII and IncN plasmid carrying *bla*_KPC−2_ while lost the IncA/C2 plasmid. The *bla*_KPC−2_-carrying plasmid pMM-R may have been acquired from another MDR organism that was not cultured. The horizontal transfer of plasmids containing resistance genes is an essential mechanism for the dispersion of antimicrobial resistance (Mathers et al., 2015). Sidjabat et al. (2009) also revealed that *bla*_KPC−2_ was likely transferred *in vivo* from *E. coli* to *Serratia marcescens* by conjugation of the plasmid harboring *bla*_KPC−2_.

Co-infections with carbapenem-resistant strains of Gram-negative species are a point of interest in medical microbiology (Marchaim et al., 2012). All patients except patient 1 had co-infection or co-colonization with carbapenem-resistant *A. baumannii* (patient 2), *P. aeruginosa* and *K. pneumoniae* (patient 3), *P. aeruginosa, A. baumannii* and *K. pneumoniae* (patient 4), respectively. Under antimicrobial selection pressure, transposition of insertion sequences or movement of plasmid from these co-infected strains may emerge as responses to environment changes (Sidjabat et al., 2009). Those co-infecting carbapenem-resistant strains were investigated to track the potential sources of *bla*_KPC−2_ genetic elements and plasmids. Clinical strains of *K. pneumoniae* from patients 3 and 4 in fact harbored *bla*_*KPC*−2_; however, the sizes of the plasmids harboring *bla*_KPC−2_ were not identical to the plasmids from pMM-R and pEA-R, respectively (data not shown). Although the source of plasmids harboring *bla*_KPC−2_ could not be identified, we assume that *bla*_KPC−2_ was acquired from the co-infecting or co-colonizing strains. The mobility of *bla*_KPC−2_ genetic elements may be responsible for the formation of plasmids pMM-R and pEA-R.

In conclusion, our results suggest that, in addition to clonal expansion, mobile genetic elements including insertion sequences and plasmids can play a significant role in the acquisition of carbapenem resistance *in vivo*, adding further complexity to the already complicated molecular epidemiology.

## Author contributions

BD and ZS involved acquisition of data (laboratory and clinical), data analysis and interpretation, drafting of manuscript and critical revision. They contributed equally for this work. FH, MY, XX, and QG participated this work for acquisition of data, discussion, and manuscript revision. MW designed this work and had critical manuscript revision.

### Conflict of interest statement

The authors declare that the research was conducted in the absence of any commercial or financial relationships that could be construed as a potential conflict of interest.
