# Supplementary material for: In vivo Acquisition of Carbapenemase Gene blaKPC-2 in Multiple Species of Enterobacteriaceae through Horizontal Transfer of Insertion Sequence or Plasmid
Source: Front Microbiol. 2016 Oct 21;7:1651. doi: 10.3389/fmicb.2016.01651 (PMC5073136; doi:10.3389/fmicb.2016.01651)
Supplement: Supplementary file 1 [file DataSheet1.DOC]

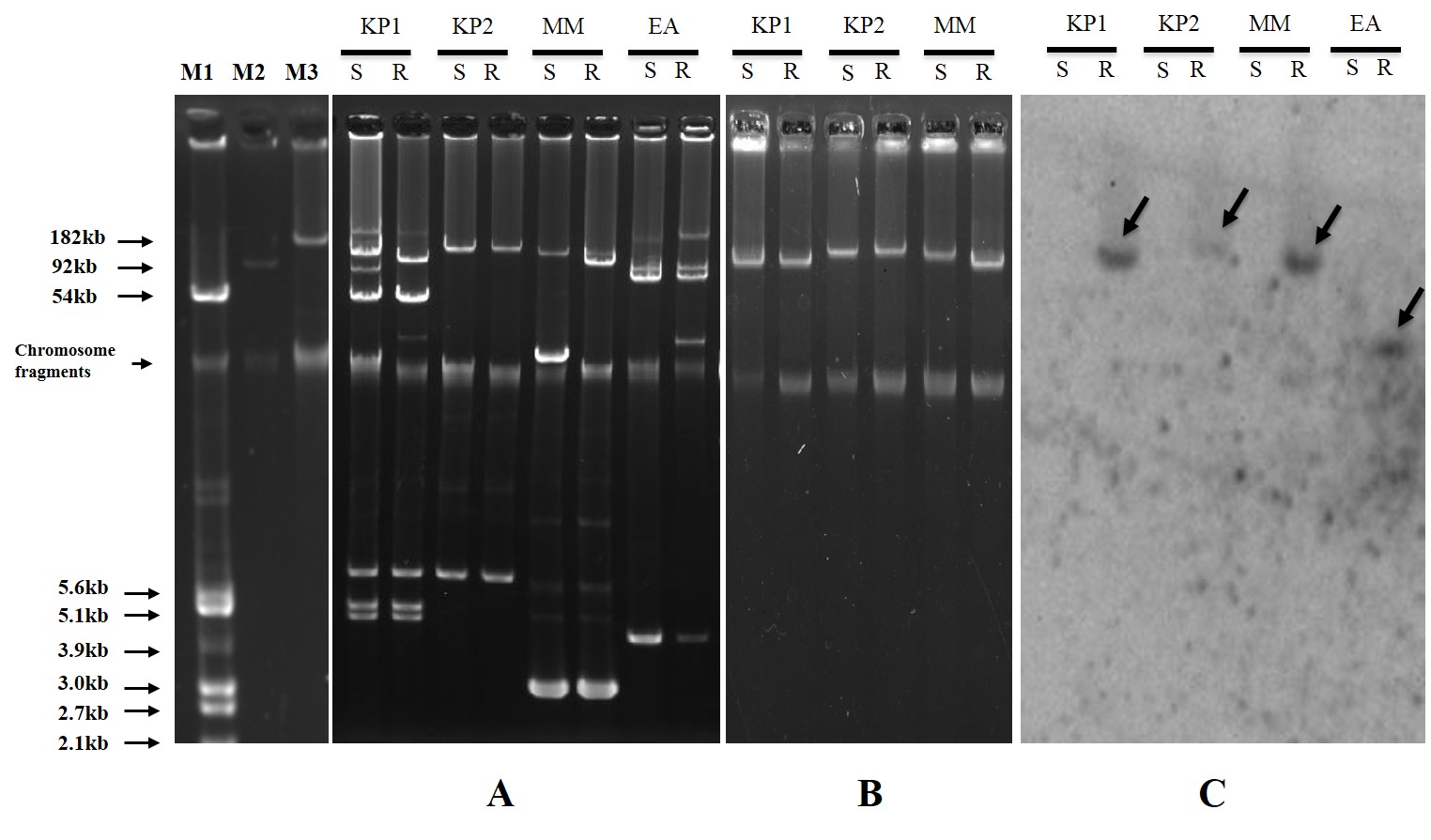


Supplementary FIGURE 1. Electrophoresis patterns of plasmids of clinical isolates and transformants and southern-blot analysis of *bla*_KPC_ harboring plasmids. Lane M1, *E. coli* V517; lane M2, *E. coli* J53 R1; lane M3, *E. coli* J53 R27. (A) Plasmids of clinical isolates. (B) Plasmids from transformants of clinical isolates. (C) Southern-blot analysis of *bla*_KPC_ harboring plasmids of clinical isolates. The arrows referred to the bands of *bla*_KPC_ harboring plasmids. KP, *Klebsiella pneumoniae*; MM, *Morganella morganii*; EA, *Enterobacter aerogenes*; S, susceptible; R, resistance.
